# Silicon and Carbon Nanocomposite Spheres with Enhanced Electrochemical Performance for Full Cell Lithium Ion Batteries

**DOI:** 10.1038/srep44838

**Published:** 2017-03-21

**Authors:** Wei Wang, Zachary Favors, Changling Li, Chueh Liu, Rachel Ye, Chengyin Fu, Krassimir Bozhilov, Juchen Guo, Mihrimah Ozkan, Cengiz S. Ozkan

**Affiliations:** 1 Materials Science and Engineering Program, University of California, Riverside, CA 92521, USA; 2 Department of Mechanical Engineering, University of California, Riverside, CA 92521, USA; 3 Department of Chemical and Env. Engineering, University of California, Riverside, CA 92521, USA; 4 Central Facility for Microscopy and Microanalysis, University of California, Riverside, CA 92521, USA; 5 Department of Electrical and Comp. Engineering, University of California, Riverside, CA 92521, USA

## Abstract

Herein, facile synthesis of monodisperse silicon and carbon nanocomposite spheres (MSNSs) is achieved via a simple and scalable surface-protected magnesiothermic reduction with subsequent chemical vapor deposition (CVD) process. Li-ion batteries (LIBs) were fabricated to test the utility of MSNSs as an anode material. LIB anodes based on MSNSs demonstrate a high reversible capacity of 3207 mAh g^−1^, superior rate performance, and excellent cycling stability. Furthermore, the performance of full cell LIBs was evaluated by using MSNS anode and a LiCoO_2_ cathode with practical electrode loadings. The MSNS/LiCoO_2_ full cell demonstrates high gravimetric energy density in the order of 850 Wh L^−1^ with excellent cycling stability. This work shows a proof of concept of the use of monodisperse Si and C nanocomposite spheres toward practical lithium-ion battery applications.

The increased demand for portable electronics by consumers and professionals alike has not only pushed the limits of electronic devices but also has concurrently increased the energy demand of the devices. From smartphones, tablets and up to electric vehicles and solar farms, the need for high energy rechargeable batteries is greater than ever. Rechargeable Lithium-ion batteries (LIBs) are widely applied in daily applications such as portable electronic devices and low-emission environmental friendly electric vehicles (EVs) because of their relatively high balanced specific energy and power, long cycling stability and low manufacturing cost^1^,^2^,^3^. A few extra merits enable lithium-ion battery (LIB) to be an ideal source of energy for commercial portable electronics. Firstly, lithium by nature is the lightest metal, and it has the most electropositivity. Secondly, LIBs demonstrate better safety performance compare with Li metal batteries and it offers a balanced large volumetric energy density (Wh/L) and gravimetric energy density (Wh/kg) simultaneously^1^,^2^,^3^. However, the energy density of conventional graphite-based lithium ion battery cells is greatly limited because the stoichiometric limit of Li^+^ intercalation in LiC_6_ restricts the theoretical capacitance value of graphite to be about 372 mAh g^−1^ (about 837 mAh cm^−3^)^4^. Though carbon based nanomaterials such as 1D CNT^5^,^6^, 2D graphene^7^,^8^, and 3D activated and template-derived carbon^9^ have lately been found to boost the anode capacity, the anode capacity is still mostly limited to be below 1000 mAh g^−1^. Also, silicon (Si) is considered and has proven to be a more promising anode material due to its highest known theoretical capacity value of 3572 mAh g^−1^ corresponding to the formation of Li_15_Si_4_ phase under ambient temperature^10^,^11^. However, silicon-based anodes suffer huge volume expansion, upwards of up to 300% during the lithiation process which induces uneven stress-strain distribution within the particle and causes pulverization and loss of active material. To remedy the aforementioned issue of anode pulverization, significant academical and industrial efforts have been made on the synthesis of nano silicon, development of novel binder systems and the design of novel nanostructured Si anode materials^3^,^12^,^13^,^14^,^15^,^16^,^17^. 3D porous Si structures demonstrate stable cycling due to the large electrolyte accessible surface area, shorter Li-ion diffusion length, and high electron conductivity^18^,^19^,^20^,^21^,^22^. However, the aforementioned porous nano silicon is mostly produced via etching of Si wafers or other doped Si materials, which require very expensive raw materials and high processing cost. Another detrimental factor that limits the application of porous and nano silicon anodes in full cell applications is its high surface area. The formation and build up of a solid electrolyte interface (SEI) layer on large surface area Si materials consumes lithium, which inturn causes huge irreversible capacity loss. Previously, we reported the synthesis of monodisperse porous silicon nanospheres (MPSSs) via a simple and scalable hydrolysis process with subsequent surface-protected magnesiothermic reduction^21^. The monodisperse and spherical nature of the MPSSs allows for a homogeneous stress-strain distribution within the particle during lithiation and delithiation, which dramatically improves the electrochemical stability. However, like most other porous nano silicon materials, MPSSs have relatively larger irreversible capacities because of the relatively larger surface area^21^,^22^. In addition, the incompatibility of conventional micrometer level carbon black within the MPSS anodes causes the MPSSs have the low reversible capacity and poor coulombic efficiency under high rates (1 C or 2 C). Though in the previous study, the addition of a certain amount of carbon nanotubes (CNTs) had been verified to be effective to improve the rate performance and cycling stability without changing the active materials ratio. The high cost of CNTs and poor coulombic efficiency of MPSS still limit their application in battery full cells^21^.

In this work, we report an innovative and facile synthesis of monodisperse silicon and carbon nanocomposite spheres (MSNSs) via a simple and scalable surface-protected magnesiothermic reduction process with subsequent chemical vapor deposition. The MSNS has several advantages. (1) The MSNSs still preserve the monodisperse spherical nature which allows a homogeneous stress-strain distribution within the structure during lithiation and delithiation. (2) The MSNS demonstrates much higher (around 25% increase) initial coulombic efficiency of 71.3% (vs. MPSS is 57.25%). (3) Li-ion battery anodes based on MSNSs demonstrate a higher reversible capacity of 3207 mAh g^−1^ compared with previously reported MPSSs anodes, superior rate performance, and enhanced cycling stability under near full utilization of anodes. As a proof-of-concept for practical LIB applications, full cells with MSNS as anode and lithium cobalt oxide (LiCoO_2_) as a cathode were fabricated. The MSNS/ LiCoO_2_ full cell is operated between 3.3 and 4.3 V delivers a high reversible capacity of 3.52 mAh cm^−2^, with a measured high energy density on the order of 850 Wh/L with the consideration of both cathodes and anodes. This value can be further boosted by optimizing the electrode structure and cell balancing. We believe this MSNS design could open new opportunities in high energy density LIBs.

## Results

**Figure 1:**
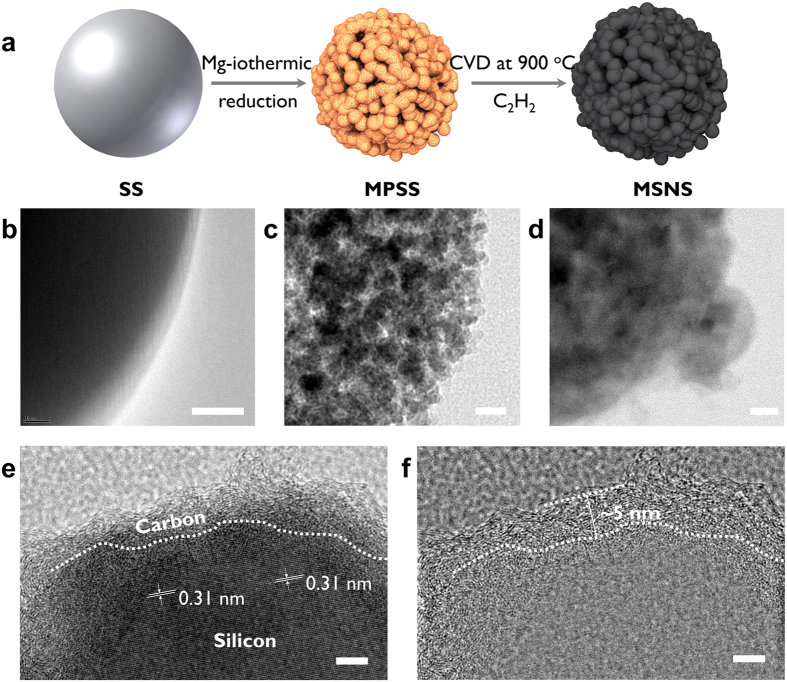
(**a**) Schematic illustration of monodisperse Si-C composite nanosphere (MSNS) formation through surface-protected magnesiothermic reduction with subsequent chemical vapor deposition (CVD). Transmission electron microscopy (TEM) micrograph of (**b**) silica sphere (SS), (**c**) monodisperse porous silicon sphere (MPSS) and (**d**) monodisperse Si-C composite nanosphere. Scale bar: 20 nm. (**e**) High-resolution TEM micrograph of MSNS and (**f**) High-resolution TEM micrograph of MSNS after FFT filtering of silica. Scalebar: 5 nm.

Figure 1a Shows the detailed schematic illustration of the synthesis process of MSNS. The MSNS is obtained via a facile surface-protected magnesiothermic reduction process with subsequent chemical vapor deposition. Firstly, gram-level monodisperse solid silica nanospheres (SSs) were prepared via a modified Stober method (Fig. 1b)^23^. The size of the as-synthesized SSs is controllable within the range of 0.05 μm–2 μm. In this work, the size of the starting material (SS) is around 0.2 μm. Adapted the previously proposed idea to preserve the size and shape of the nanospheres, sodium chloride (NaCl) was introduced as a safe, economical and efficient heat scavenger^24^,^25^. The local melting of Si and, consequently, aggregation of nano-Si particles can be caused by the magnesiothermic reduction process since it evolves a significant amount of heat^26^. However, by surrounding the as-prepared SSs with the optimum amount of NaCl the fusion of Si can be minimized. The premixed SS, NaCl, and Mg powders are heated up to 700 °C to trigger the reduction, as in Equ. 1.


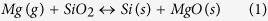



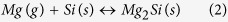


Unwanted product magnesium silicide (Mg_2_Si) can result from excess Mg alloying with Si, as in Eq. 2. The undesired Mg_2_Si and excessive NaCl can be simply removed by repeatedly washing with deionized (D.I.) water and HCl acid. Transmission electron microscopy (TEM) micrographs are shown to present the detailed morphology and structural evolution during the synthesis process (Fig. 1b–d). TEM image shows the well-preserved monodisperse nature while also showing the as-synthesized MPSSs are highly porous, with pore diameter in the range of 10–30 nm (Fig. 1c; for details see Fig. S2). Porous silicon nanostructures have been proved to have low stress during lithiation and delithiation, which helps to maintain its structural integrity after cycling^26^. Instead of using pure lithium as the counter electrode in half-cell configuration, practical LIBs normally use lithium transition metal oxides as cathodes which have limited lithium ions within the cell. Anodes with very high surface areas, especially Si-based anodes, are not desirable because of their relatively high irreversible capacity loss, due to the formation and buildup of the solid electrolyte interface (SEI) layer. To remedy this problem, in this work, we propose to modify the porous MPSSs with carbon to form a monodisperse silicon and carbon nanocomposite sphere structure via a simple and scalable CVD process. The CVD carbon modification is achieved via cracking of C_2_H_2_ under ambient pressure at 900 °C. Transmission electron microscopy (TEM) image shows the MPSSs were successfully coated with carbon after CVD process and the mesopores within the MPSS structure were filled with graphitic carbon materials (Fig. 1d, for details, see Fig. S2). High-resolution TEM images are shown to further characterize the carbon and silicon interface and the uniformity of the carbon layer. High-resolution transmission electron microscopy (HRTEM) image together with the corresponding FFT suggests the silicon within the MSNSs are crystalline with clear lattice fringes corresponding to Si (111), which shows a d spacing of 0.31 nm (Fig. 1e, Fig. S1a). The d-spacing of carbon (0.35 nm) and polycrystalline silicon (0.31 nm) is too close, to better distinguish the carbon and silicon interface and confirm the thickness of the carbon coating layer, HRTEM and corresponding inverted FFT images are collected (Fig. S1a,b). The Fourier masked micrographs of Fig. S1a reveals the carbon layer is uniformly coated with a thickness around 4–5 nm which corresponds to ~10 carbon layers (Fig. 1f and Fig. S1c,d).

**Figure 2:**
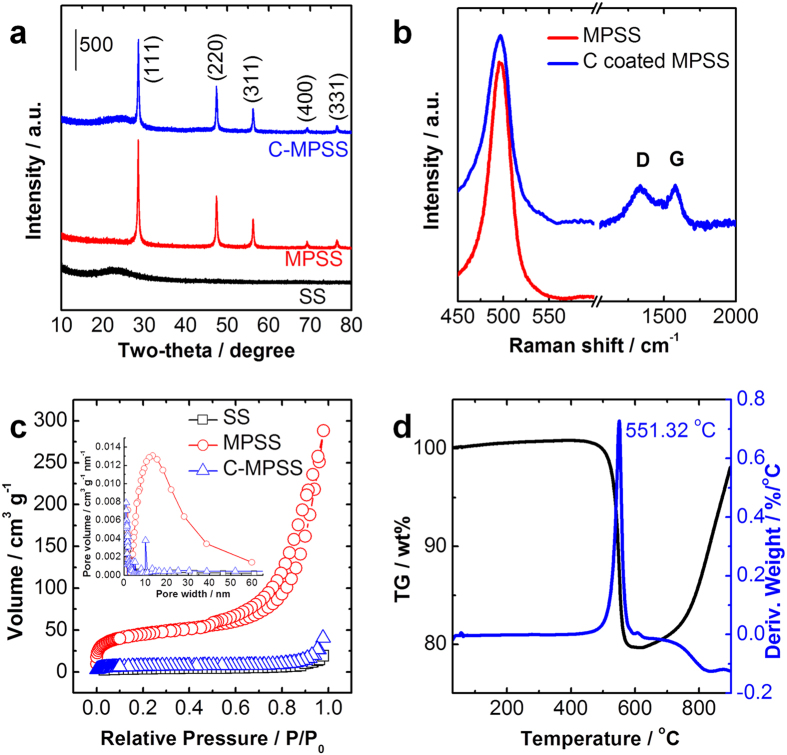
(**a**) XRD patterns of SS, MPSS and MSNS. (**b**) Raman spectra of MPSS and MSNSS. (**c**) BET surface area measurements of SS, MPSS, and MSNS with type IV N_2_ adsorption and desorption isotherms. The inset shows the pore size distribution of SS, MPSS, and MSNS. (**d**) TGA/DTA analysis of the MSNS between room temperature and 900 °C in air. Scan rate: 10 °C/min.

Powder X-ray diffraction (XRD) was conducted to characterize the crystallinity and purity of the starting material SSs and as-synthesized MPSSs and MSNSs, (Fig. 2a). The black XRD pattern is consistent with typical amorphous phase structure of silica materials. The red and blue XRD patterns can be indexed as polycrystalline silicon while the spectra of MSNSs (blue) also shows one broad peak around 25 degree, which can be due to the existence of graphitic carbon in the MSNSs. The narrow and sharp XRD peaks absence of obvious amorphous scattering background exhibited by MPSSs and MSNSs suggest a high degree of crystallinity, and they can be indexed to a cubic phase of silicon. To further examine the as obtained MPSSs and MSNSs material, Raman spectroscopy was performed (Fig. 2b). The sharp peaks at a shift of 521 cm^−1^ for both MPSS and MSNS are in good agreement with the Raman spectra of polycrystalline silicon which is in accordance with the findings from XRD patterns shown in Fig. 2a. Besides the peak at a shift of 521 cm^−1^, the MSNS shows an additional D peak centered around 1340 cm^−1^ and the G peak centered around 1572 cm^−1^, which further confirmed the carbon and silicon nanocomposite nature of the MSNSs. The presence of the intense D band in the spectrum is typically associated with disorder or defects in nanostructured carbonaceous materials, and the MSNS shows high I_D_/I_G_ (~1) suggests that the MSNSs have high level of disorder^27^,^28^,^29^,^30^,^31^,^32^,^33^,^34^. We believe disorders of carbon coating of MPSS can be beneficial in this work since it could offer better ion permeability and help to limit the outward expansion during electrochemical charge and discharge process. HRTEM images in Fig. 1b,c have evidently shown the SSs, MPSSs, and MSNSs have distinctly different porosity. Nitrogen adsorption isotherms are shown to confirm further the porosity and surface area evolution from solid to mesoporous and to solid for SS, MPSS, and MSNS, respectively. The MPSSs show a nearly 20 times enlargement in surface area (Langmuir: 353.22, Brunauer-Emmett-Teller (BET): 214.65 m^2^ g^−1^) compare with SSs (Langmuir: 20.07, BET:11.89 m^2^ g^−1^) after Mg reduction as suggested by our former study (Fig. 2c)^21^. It is interesting to note that the Langmuir and BET surface areas of MSNSs are reduced to34.64 m^2^ g^−1^ and 20.94 m^2^ g^−1^, respectively after CVD carbon coating process which suggests the final product has limited pores. The inset of Fig. 2c shows the pore distribution of SS, MPSS, and MSNS, which suggests MPSS possesses very high surface area due to a combination of mesopores and micropores while both SSs and MSNSs have limited surface area and porosity. Thermogravimetric analysis (TGA) shown in Fig. 2d indicates the silicon content in MSNSs is around 80% (Fig. 2d).

**Figure 3:**
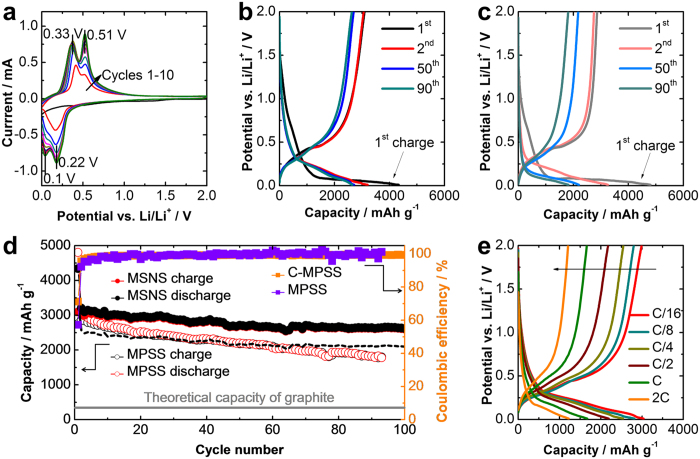
(**a**) Cyclic voltammetry characteristic of the MSNS based LIB anodes. Scan rate: 0.2 mV sec^−1^. (**b,c**) 1^st^, 2^nd^, 50^th^, 90^th^ galvanostatic charge-discharge profiles of MSNS and MPSS respectively. (**d**) Cycling performance and coulombic efficiency of the MSNS and MPSS electrodes at a current density of C/20^21^. (**e**) Galvanostatic charge-discharge profiles of MSNS electrodes under different rates of charge and discharge.

**Figure 4:**
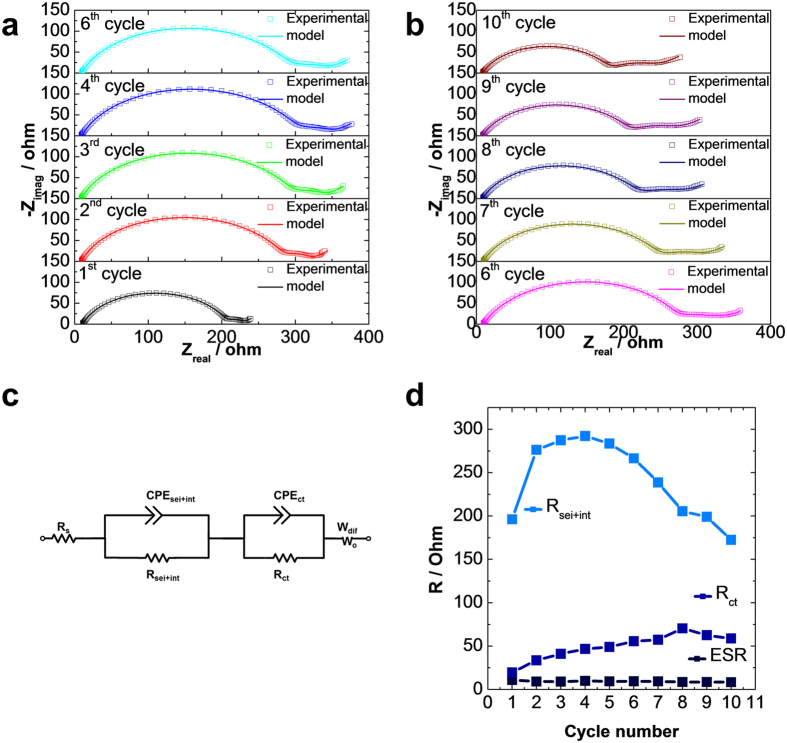
(**a**,**b**) Experimental and model fitted EIS plots of the MSNS electrodes. (**c**) The equivalent circuit used to model the EIS spectra. (**d**) Equivalent series resistance, SEI and interphase electronic contact resistance, and charge transfer resistance as a function of cycling number.

Coin half cells were built to evaluate the electrochemical performance of the MSNSs as anode active material. 2032-type coin half cells were built in an Ar-filled glove box with moisture and oxygen level below 0.5 ppm. MSNS electrode was used as the anode, and pure Li metal chip was as the counter electrode. The electrodes were prepared by mixing 90% active material (MSNS in this work) with 10% alginate binder, and the as-prepared anode slurry was cast on Cu foil Fig. 3a shows the cyclic voltammogram of the MSNS anode half cells at a scan rate of 0.2 mV sec^−1^. It is promising to see no noticeable peaks observed during first charge process for above0.5 V region which suggests a very low irreversible charge capacity in the first cycle. We believe this is due to much lower level of solid-electrolyte-interface (SEI) layer formation and buildup (0.5–0.7 V) for MSNS electrode compare with previously reported MPSS electrode^21^. The two broad lithiation peaks observed during charge process can be attributed to the electrochemically-driven solid-state alloying Li_x_Si (if it is fully charged, x ≈ 3.75), while the delithiation peaks during discharge process are from dealloying of amorphous lithium silicide (a-Li_x_Si, x ~ 3.75)^3^,^35^. After the formation and activation in the first three cycles, the current-potential characteristics become approximately consistent for the cycles afterward. The voltage profiles are observed to be in good agreement with the cyclic voltammograms. Figure 3b shows the 1^st^ charge, 2^nd^, 50^th^, and 90^th^ cycle voltage profiles of MSNS based coin half cell. The cells were deep charged and discharged between 0.01 V and 2.0 V at a rate of C/20. Compare with our previously reported MPSS anode, a relatively higher reversible discharge capacity of 3207 mAh g^−1^ is achieved by the MSNS cell (MPSS cell: 3105 mAh g^−1^ at C/20) from the 2^nd^ cycle (after the first charge). Besides higher reversible capacity, near consistent charge-discharge characteristics are observed for MSNS cell in subsequent cycles. (50^th^ cycle: 2715 mAh g^−1^, 90^th^ cycle: 2645 mAh g^−1^). The capacity faded relatively faster for MPSS anode during the subsequent cycles compared with MSNS anode (50^th^ cycle: 2180 mAh g^−1^, 90^th^ cycle: 1823 mAh g^−1^). In addition, the MSNS electrode demonstrates much lower irreversible capacity under deep charging and discharging in the first cycle (Fig. 3b,c). The C/20 cycling performance of MSNS and MPSS electrodes is demonstrated in Fig. 3d. An over 85% capacity retention is achieved by MSNS cell over 100 charge-discharge cycles at relatively low rate of C/which is much higher than the MPSS cell^21^. Coulombic efficiency (CE) is a critical factor to judge the reversibility of the cell. After the SEI formation in the first cycle, the CE of MSNS anode half cells obtained for all cycles is around 100%, suggesting MSNS based anodes have excellent reversibility. The discharge irreversible capacity for the 1^st^ charge is due to the formation of the SEI layer on the surface of electrodes^5^. For full cell LIBs, which normally use lithium transition metal oxide as a cathode, the initial coulombic efficiency is essential. It is evident that the MSNS demonstrates much higher initial coulombic efficiency (ICE) of 71.3% (vs. MPSS is 57.25%). The dashed line in Fig. 4d shows the capacity retention vs. cycling based on the total weight of silicon and carbon in the MSNS structure which is around 80% of the capacities calculated based on silicon only. Figure 4d suggests even taking the weight of conductive additive into account; the MSNS system still demonstrates a comparable reversible capacity and superior cycling stability. The rate capability of the MSNS anode was evaluated via using galvanostatic cycling under various rates of C/16, C/8, C/4, C/2, C, and 2 C (Fig. 3d). It is evident that even under high rate around 2 C (30 min charge) the anode capacity is still maintained above 1200 mAh g^−1^ which is almost three times higher than conventional graphite-based anodes.

## Discussion

To better understand the charge transfer and ion transfer mechanism of the MSNS anodes, electrochemical impedance spectroscopy (EIS) measurements were conducted for ten consecutive cycles for MSNS based anode half cells under the fully charged state. The experimental and fitted EIS plots are summarized in Fig. 4a,b which all consist of two semicircles and one near linear diffusion drift. The equivalent circuit used for fitting for our MSNS-based electrode system is shown in Fig. 4c 36,37. The fitted impedance parameters including equivalent series resistance (ESR), interfacial resistance (R_sei+int_), and the charge transfer resistance (R_ct)_ at different cycles are summarized in Fig. 4d. Equivalent series resistance (*ESR)* commonly referred to the high-frequency intercept is relates to the electronic conductivity of the electrodes, contact resistances associated with cell components, as well as the ionic conductivity of the electrolyte solution^38^,^39^. The MSNS anode half cell exhibits low and stable ESR values around 10 ohms with a small range of fluctuations (±1 ohm) in the first ten cycles. The high-frequency depressed semicircle in this work corresponds to The interfacial impedance due to the formation of solid electrolyte interface (SEI) layer^40^ and interface electronic contacts between the current collector and active material^41^,^42^ (R_sei+int_) corresponds to the high frequency depressed semicircle (100 kHz to 200 mHz)^20^,^43^. The R_sei+int_ for MSNS based LIB system increases in the first few cycles, and it tends to stabilize and then gradually decrease afterward. The R_sei+int_ increase in the first few cycles can be attributed to the formation and buildup of SEI layer. In coin cell configuration, the electrodes are pressed by a spring, the decrease of R_sei+int_ may be due to the irreversible electrode volume change during cycling which results in a gradual increase of the clamping pressure on the electrodes. Therefore, the R_sei+int_ was slightly improved. The charge-transfer resistance (*R*_*ct*_) is associated with the medium-frequency depressed semicircle^44^ The *R*_*ct*_ values for MSNS anode half cells constantly increase over the first few cycles and tend to stabilize at around 50 ohms after 8–10 cycles. The charge transfer reaction predominantly happens on the surface of the active material, in this work, the electronic contact between carbon and silicon within the MSNS nanocomposite system has a great impact on the charge transfer reaction. The carbon coating provides an interpenetrating conductive network within the MSNS nanocomposite system which facilitates charge transfer and minimizes the loss of the electronic contact between MSNS and polymer binder. The Warburg impedance (*W*_*O*_) describes diffusion-related phenomena in both electrolyte and bulk electrode which is associated with the low-frequency tail (<200 mHz)^38^,^39^. The shorter tails in the EIS plots for the MSNS anode half cells (Fig. 4a,b) implies faster and more facile diffusion of Li^+^ in the cell^21^,^38^.

**Figure 5:**
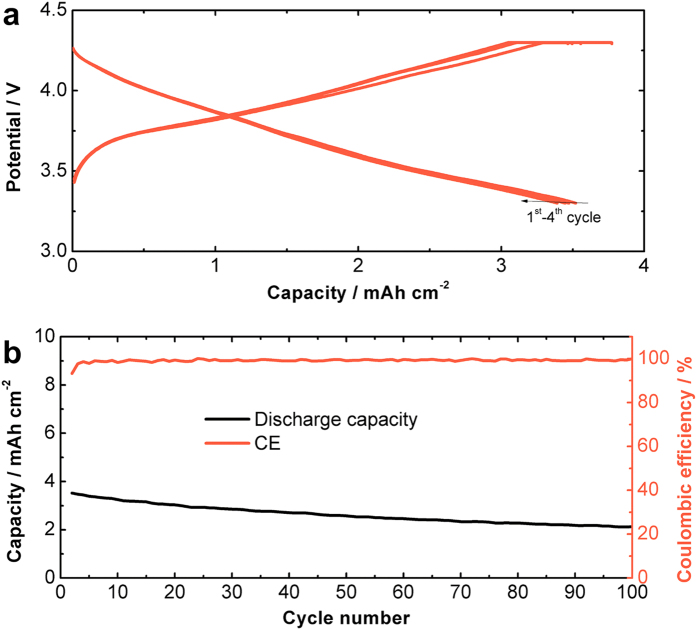
(**a**) Galvanostatic voltage profiles from the 1^st^ to the 4^th^ cycle and (**b**) the cycling response of the MSNS/LiCoO_2_ full cell at C/2 rate.

Finally, a full cell LIB is demonstrated by employing MSNS anode and LiCoO_2_ (LCO) cathode. The cell balance value (capacity ratio of the negative and positive electrode) is selected to be slightly larger than 1 to ensure the cell is cathode limited. The electrochemical performance of the MSNS/LCO full cell is studied by the galvanostatic charge and discharge cycles with an operational voltage window from 4.3 to 3.3 V. The MSNS/ LCO full cell is activated for 1 cycle at the rate of C/20 (based on the cathode capacity) and then the cell is cycled at C/2 for 100 cycles. Figure 5a shows galvanostatic voltage profiles for the 1^st^ to 4^th^ cycle. The reversible discharge capacity is measured to be 3.52 mAh cm^−2^ in the 1st cycle and the reversible capacity is maintained above 2.2 mAh cm^−2^ for the subsequent cycles with a coulombic efficiency (CE) of >99.9% (Fig. 5b). The energy density of the MSNS/LCO full cell is measured to be on the order of 850 Wh/L with the consideration of both cathode and anode, and we anticipate this value can be further increased by optimizing the electrode structure and cell balancing.

In conclusion, synthesis of monodisperse Si and C nanocomposite spheres via a facile magnesiothermic reduction with subsequent CVD process has been demonstrated. We believe the monodisperse and high symmetrical nature of the composite spheres allow a homogeneous stress-strain distribution within the structure during charge and discharge. Anode half cells based on MSNSs demonstrate a higher reversible capacity of 3207 mAh g^−1^, enhanced cycling stability, improved ICE and rate performance compare with previously reported MPSS anode system. The MSNS/LCO full cell design shows a high volumetric energy density of 850 Wh/L and excellent cycling stability. We believe optimization and further development of this MSNS anode design will lead to new opportunities for high energy density energy LIBs.

## Methods

### Synthesis of MSNS

Monodispersed solid silica nanospheres (SS) and monodisperse porous silicon nanospheres (MPSSs) are prepared via the modified Stober method and previously reported surface protected magnesiothermic reduction, respectively^21^,^23^. SS powder is milled with NaCl in a 1:10 w/wand then the SS/NaCl mixture is immersed in deionized water under ultrasonication and stirring for 1 hour. Well mixed SS/NaCl powder is achieved by removing water by drying. Then the SS/NaCl powder is mixed with Mg powder (99.5%, −325 mesh, Sigma-Aldrich) in a 1:0.9 w/w SS: Mg ratio. Next, the SS/NaCl/Mg mixture is heated to 700 °C at a ramping rate of 5 °C/min, held at 700 C for 6 hours, and cooled to room temperature in the inert environment. The NaCl is removed from resulting product by washing with water several times. Unwanted Mg_2_Si and MgO are removed via etching in concentrated HCl overnight with subsequent washing with DI H_2_O. Unreacted SiO_2_ is removed by HF etching. The rinsed powder is dispersed in ethanol and ultimately dried under vacuum for 4 hours at 100 °C. To achieve MSNSs, the resulting MPSSs are heated in a hot-wall CVD furnace to 900 °C under ambient pressure in an Ar/H_2_ atmosphere, and once the temperature reaches 900 °C, acetylene (C_2_H_2_) is introduced to trigger and continue the growth of carbon layer.

### Materials characterization

The surface morphology of SS, MPSS and MSNS is examined using scanning electron microscopy (SEM; leo-supra, 1550) and transmission electron microscopy (TEM; Philips, CM300) with a LaB_6_ cathode operated at 300 KV. The crystal structure is analyzed with a PANalytical Empyrean X-Ray Diffractometer (XRD). The Raman spectra of SS, MPSS and MSNS, are collected with a Renishaw DXR Raman spectroscopy system with a 532 nm laser (8 mW excitation power, 100x objective lens). The BET surface area and pore distribution are measured by a Quantachrome BET analyzer.

### Electrochemical Tests

The MPSS electrodes were prepared by casting a slurry containing 70% active material 20% conductive additive (carbon black), and 10% sodium alginate binder^21^. The MSNS contains about 20% carbon, so the powder itself is considered conductive, the MSNS electrodes are prepared by casting a slurry containing 90% active material (MSNS) with 10 wt% sodium alginate binder without any conductive additive. The per area mass loading was 1–5 mg cm^−2^ CR 2032 coin cell configuration is used for the electrochemical measurements. The cells are assembled in an Ar-filled glove box. Pure Li metal chip is used as the counter electrode for coin half cells. Commercial LiCoO_2_ cathodes (provided by Temiz Energy Technologies) are utilized for the fabrication of coin full cell. Celgard 3501 porous membrane is used as the separator. The electrolyte employed in this work is 1 M LiPF_6_ dissolved in a 1:1 (by volume) mixture of ethylene carbonate (EC) and dimethyl carbonate (DMC). Cyclic voltammetry scans were conducted at a scan rate of 0.2 mV sec^−1^ with an operational voltage window of 0.01 to 2.0 V (vs. potential of Li^+^/Li). Galvanostatic charge-discharge and cycling performance measurements are conducted at a fixed operational voltage window between 0.01 V and 2.0 V for anode half cells. The MSNS/LCO full cells are measured with a fixed cell voltage between 3.3 V and 4.3 V. Potentiostatic electrochemical impedance spectroscopy (EIS) analysis was conducted between 0.01 Hz and 1 MHz with an amplitude of 10 mV under 100% state of charge (SOC).

## Additional Information

**How to cite this article:** Wang, W. *et al*. Silicon and Carbon Nanocomposite Spheres with Enhanced Electrochemical Performance for Full Cell Lithium Ion Batteries. *Sci. Rep.* **7**, 44838; doi: 10.1038/srep44838 (2017).

**Publisher's note:** Springer Nature remains neutral with regard to jurisdictional claims in published maps and institutional affiliations.

## Supplementary Material

Supplementary Information
