# Supplementary material for: Silicon and Carbon Nanocomposite Spheres with Enhanced Electrochemical Performance for Full Cell Lithium Ion Batteries
Source: Sci Rep. 2017 Mar 21;7:44838. doi: 10.1038/srep44838 (PMC5359662; doi:10.1038/srep44838)
Supplement: Supplementary Information [file srep44838-s1.doc]

supporting information for

Subject areas

Lithium-ion Batteries, Silicon, Anode, Energy Storage, Sphere

Correspondence and requests for materials should be addressed to

C. S. O. ([cozkan@engr.ucr.edu](mailto:cozkan@engr.ucr.edu))

**Silicon and Carbon Nanocomposite Spheres with Enhanced Electrochemical Performance for Full Cell Lithium Ion Batteries**

Wei Wang^1^, Zachary Favors^1^, Changling Li^1^, Chueh Liu^1^, Rachel Ye^2^, Chengyin Fu^3^, Krassimir Bozhilov^4^, Juchen Guo^1,3^, Mihrimah Ozkan^1,5^ and Cengiz S. Ozkan^1,2*^

^1^Materials Science and Engineering Program, University of California, Riverside, CA 92521 USA

^2^Department of Mechanical Engineering, University of California, Riverside, CA 92521 USA

^3^Department of Chemical and Env. Engineering, University of California, Riverside, CA 92521 USA

^4^ Central Facility for Microscopy and Microanalysis, University of California, Riverside, CA 92521 USA

^5^Department of Electrical and Comp. Engineering, University of California, Riverside, CA 92521 USA


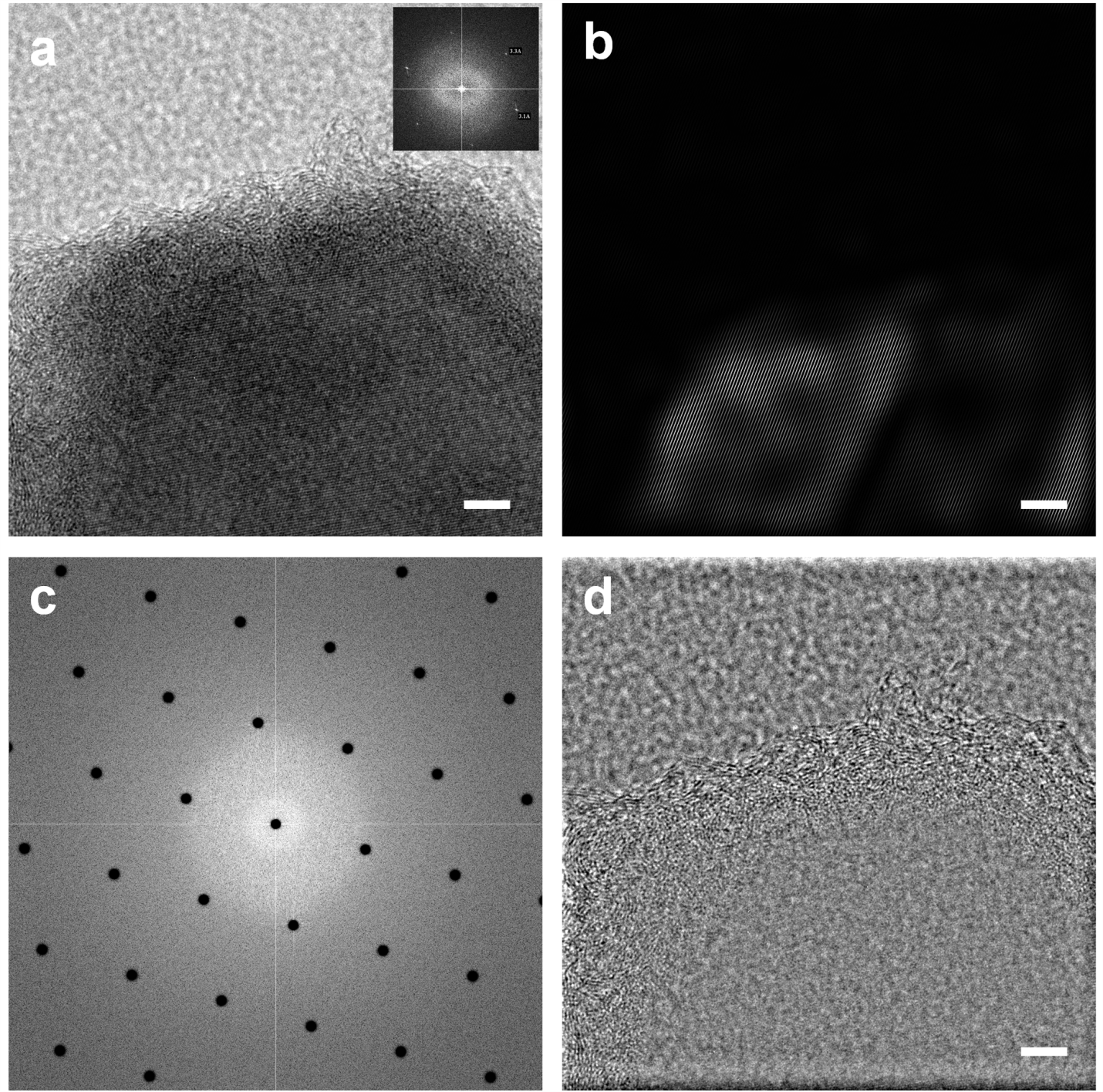


**Figure S1.** (a) HRTEM micrograph and corresponding FFT image (inset) of MSNS. (b) Inverted FFT image of MSNS. (c-d) orientation Fourier-masked micrographs of (a). Scale bar: 5nm.


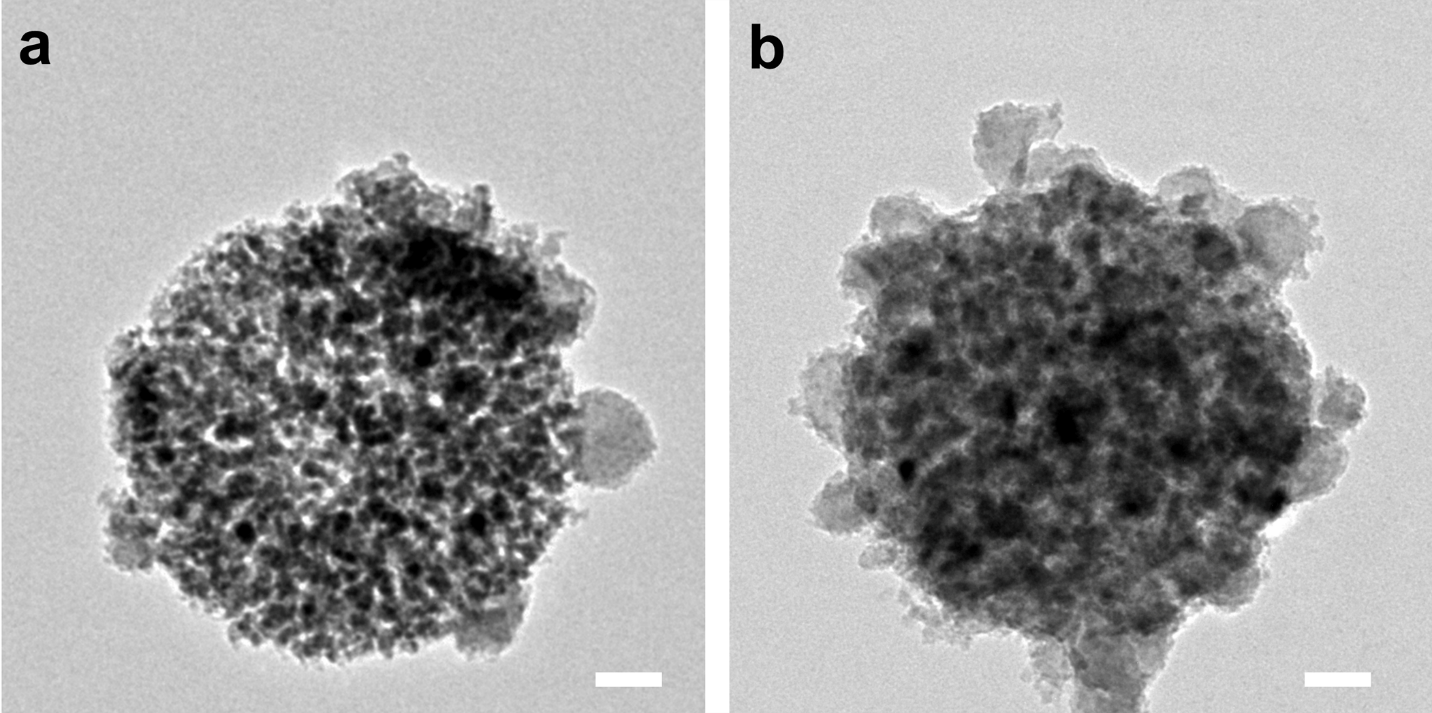


**Figure S2.** Transimission electron microscopy (TEM) micrograph of (a) monodisperse porous silicon sphere (MPSS)^1^ and (b) monodisperse Si-C composite nanosphere. Scale bar: 50 nm.


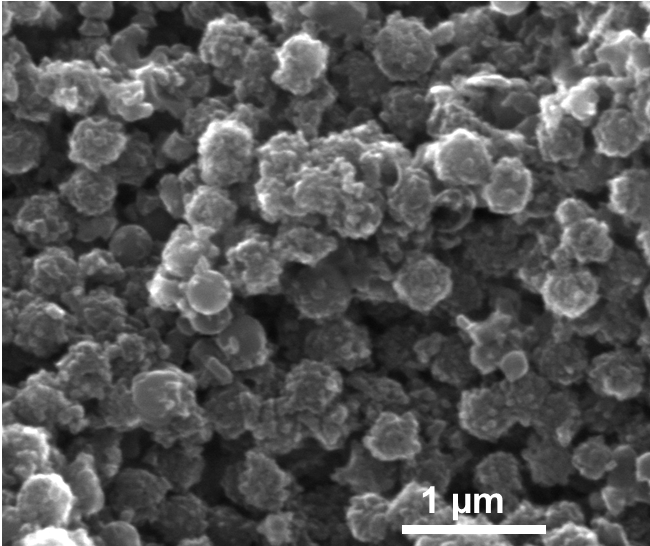


**Figure S3.** Scanning electron microscopy (SEM) micrograph of MSNS electrode.

References

1 Wang, *W. et a*l. Monodisperse porous silicon spheres as anode materials for lithium ion batteries*. Scientific repor*t**s** 5 (2015).
